# Designing a T-cell epitope-based vaccine using in silico approaches against the Sal k 1 allergen of *Salsola kali* plant

**DOI:** 10.1038/s41598-024-55788-x

**Published:** 2024-02-29

**Authors:** Mohammad Hossein Shams, Seyyed Mohsen Sohrabi, Reza Jafari, Ali Sheikhian, Hossein Motedayyen, Peyman Amanolahi Baharvand, Amin Hasanvand, Ali Fouladvand, Mohammad-Ali Assarehzadegan

**Affiliations:** 1 Hepatitis Research Center and Department of Medical Immunology, School of Medicine, Lorestan University of Medical Sciences, Khorramabad, Iran; 2 Department of Production Engineering and Plant Genetic, Faculty of Agriculture, Shahid Chamran University of Ahvaz, Box 6814993165, Ahvaz, Iran; 3 School of Allied Medical Sciences, Shahroud University of Medical Sciences, Shahroud, Iran; 4 Autoimmune Diseases Research Center, Kashan University of Medical Sciences, Kashan, Iran; 5 Department of Physiology and Pharmacology, School of Medicine, Lorestan University of Medical Sciences, Khorramabad, Iran; 6 Immunology Research Center, Department of Immunology, School of Medicine, Iran University of Medical Sciences, Tehran, Iran

**Keywords:** In silico, Immunoinformatics, Vaccine, T-cell epitope-based vaccine, *Salsola kali*, Sal k 1, Allergen-specific immunotherapy, Immunotherapy, Vaccines

## Abstract

Allergens originated from *Salsola kali* (*Russian thistle*) pollen grains are one of the most important sources of aeroallergens causing pollinosis in desert and semi-desert regions. T-cell epitope-based vaccines (TEV) are more effective among different therapeutic approaches developed to alleviate allergic diseases. The physicochemical properties, and B as well as T cell epitopes of Sal k 1 (a major allergen of *S. kali*) were predicted using immunoinformatic tools. A TEV was constructed using the linkers EAAAK, GPGPG and the most suitable CD4^+^ T cell epitopes. RS04 adjuvant was added as a TLR4 agonist to the amino (N) and carboxyl (C) terminus of the TEV protein. The secondary and tertiary structures, solubility, allergenicity, toxicity, stability, physicochemical properties, docking with immune receptors, BLASTp against the human and microbiota proteomes, and in silico cloning of the designed TEV were assessed using immunoinformatic analyses. Two CD4^+^ T cell epitopes of Sal k1 that had high affinity with different alleles of MHC-II were selected and used in the TEV. The molecular docking of the TEV with HLADRB1, and TLR4 showed TEV strong interactions and stable binding pose to these receptors. Moreover, the codon optimized TEV sequence was cloned between *Nco*I and *Xho*I restriction sites of pET-28a(+) expression plasmid. The designed TEV can be used as a promising candidate in allergen-specific immunotherapy against *S. kali*. Nonetheless, effectiveness of this vaccine should be validated through immunological bioassays.

## Introduction

Allergic diseases are among prevalent global health problems^1^. More exactly, more than one-third of the world's population experiences type-I allergic reactions^2^. Due to the complex pathophysiology of allergic diseases, these diseases cannot affect all people^3^. In other words, various factors such as genetic predisposition, climate change, industrialization, and route of exposure are effective in causing these diseases^4^. Aeroallergens, especially pollens, are common causes of respiratory allergies such as allergic rhinitis and asthma^5^. Allergens originated from *Salsola kali* (*Russian thistle*) pollen grains are one of the most important sources of aeroallergens causing pollinosis in desert and semi-desert regions^6,7^. Seven allergens (Sal k 1 to Sal k 7) have been identified from *Salsola kali* and recorded in the allergen nomenclature database (http://www.allergen.org) so far. It has been reported that Sal k 1 with multiple isoforms, which is the main allergen of *S. kali*, causes more than 80% of sensitization in *S. kali* allergic patients^8,9^.

Allergen specific immunotherapy (AIT) with natural allergen sources is still considered as a successful therapeutic procedure for allergies to common allergen sources such as grass pollens, house dust mites, and bee venom^10^. There are certain challenges regarding the standardization of allergen sources that have adverse side effects^11,12^. Moreover, recombinant allergens are increasingly used for diagnostic and therapeutic purposes, and innovative strategies for AIT such as DNA-based vaccines or T cell or B cell targeted therapies^10,13,14^ have been developed. Thus, determination of the molecular factors effective in immunogenicity and allergenicity of allergens has drawn the attention of researchers. Application of T-cell epitope-based vaccines (TEV) has had successful trials as a safe method in connection with Japanese cedar pollens and grass pollens, honeybee venom, and house dust mite^15,16^. In this therapy, allergen-derived soluble peptides with immunodominant T-cell epitopes are utilized to treat allergic patients^17^. It has been reported that the use of TEV is more effective than other therapeutic approaches for the treatment of allergic diseases due to the effective reduction of adverse effects and their inability to bind to IgE- FcεRI on effector cells, mainly because of the small peptide size of T-cell epitopes (10–17 amino acids)^18,19^. Thus, in the present study, we intended to predict B and helper T lymphocyte (HTL) epitopes of Sal k 1 allergen by an in-silico approach. After identifying linear B and HTL epitopes by immunoinformatic tools, the best peptides containing HTL epitopes and lacking any B cell epitopes was chosen and used in designing the TEV.

## Materials and methods

### The protein sequence retrieval and phylogenetic analysis

The protein sequences of four isoforms or variants of Sal k 1 with accession numbers P83181, AAT99258, AAX11262, and AAX11262 were obtained from the International Union of Immunological Societies nomenclature database (IUIS) (http://www.allergen.org) and the protein database of the National Center for Biotechnology Information (NCBI) (https://www.ncbi.nlm.nih.gov/). The obtained protein sequences were aligned using CLC Genomics Workbench 3.0 software, and the consensus protein sequence was employed as the BLASTp query to find homologous sequences. Investigation of the family classification of Sal k 1 was carried out using Pfam v29.0 and InterPro v56.0^20^. The TMHMM server 2.0 was used to predict the transmembrane helices of the Sal k 1^21^. The phylogenetic tree was made according to the Jones–Taylor–Thornton (JTT) model using the maximum likelihood (ML) approach in MEGA-7 software^22^. The reliability of the phylogenetic tree of the Sal k 1 amino acid sequence was computed using the bootstrap method with 1000 replications^23^.

### Physicochemical analysis and secondary structure prediction

The ExPASy ProtParam tool with default parameters was used to calculate the physicochemical characteristics of Sal k 1 protein^24^. The presence of functional motifs of the Sal k 1 was analyzed using the Prosite database^25^. Also, by NETPhos v 3.1 phosphorylation motifs were analyzed^26^. The secondary structure properties of Sal k 1 was predicted using PSIPRED and NetSurfP ver. 1.1^27,28^.

### The linear B lymphocyte epitopes prediction

The B -lymphocyte epitopes in the Sal k 1 protein were predicted using immune epitope database (IEDB) and BCepred tools. These two servers identify B lymphocyte epitopes by the physicochemical characteristics of the amino acid sequence^29^. Epitopes that have the features of accessibility, flexibility, antigenicity and hydrophobicity were selected as the main epitopes of B lymphocytes^29^. The epitopes confirmed by both IEDB and BCepred tools were chosen as the final B lymphocytes epitopes.

### The helper T lymphocyte (HTL) epitopes prediction

The prediction of 15 amino acids long HTL epitopes was performed using the MHC II prediction tool in the IEDB. Epitopes characterized by low percentile rank and SMM less than IC_50_ were considered as high affinity peptides for binding to the MHC-II molecule^30^.

### Population coverage

Population coverage for the predicted HTL epitopes is determined for world population using the population coverage calculation tool in IEDB. This tool calculates the portion of individuals predicted to react to a given set of epitopes with recognized MHC limitations^31^.

### Molecular docking of the predicted epitopes

The prediction of the 3D structure of the selected epitopes of HTL was carried out using the peptide structure prediction tool (PEP-FOLD 3)^32^. The protein–protein docking between the crystalized structure of the most abundant MHC-II allele reacting with Sal k 1 epitopes was performed in ClusPro 2.0 tool^33^. Subsequently, this molecular docking was visualized and analyzed using the PyMOL software. For this purpose, the 3D crystal structure of the most abundant MHC-II allele was retrieved from RCSPDB. Moreover, for proper docking, these 3D crystal structures were processed using UCSF Chimera software. Then, non-amino acid molecules such as ligand, ions, and solvent water were removed from it, and subsequently polar hydrogen atoms were added to it^34,35^.

### Construction of the T-cell epitope-based vaccine

In order to construct a T-cell epitope-based vaccine (TEV), the predicted HTL epitopes were fused together and to a toll-like receptor 4 (TLR4) agonist using flexible linkers. The HTL epitopes were fused using the GPGPG flexible linker^36^. A synthetic TLR4 adjuvant RS04 (Sequence: GLQQVLL) was added to the N and C-terminus of the vaccine construct using an EAAAK linker^37^. In order to find out the epitopes' identity and similarity of the constructed vaccine to proteins in humans, they were aligned against the human proteome and microbiome using the NCBI BLASTp tool^38^. The PAM30 scoring matrix and an expectation value (E-value) of 0.0001 were considered as BLASTp parameters.

### Prediction of allergenicity, toxicity, and antigenicity of the TEV

The antigenicity of the TEV sequence was predicted using the ANTIGENpro server^39^. The Algpred tool was utilized to find the allergenicity of the TEV sequence^40^. Furthermore, the ToxinPred tool was used to check the toxicity of the TEV^41^.

### Physicochemical analysis of the TEV

The Expasy ProtParam tool was used to calculate the physicochemical characteristics of the designed TEV^24^. Molecular weight, theoretical isoelectric point (pI), aliphatic index, instability index, grand average of hydropathicity (GRAVY), and in vitro as well as in vivo half-lives were among the examined properties. The SOLPro tool was used to evaluate the vaccine's solubility upon overexpression in *Escherichia coli* (*E. coli*)^42^.

### Prediction of the TEV's secondary structure

The TEV's secondary structure was anticipated using PSIPRED 4.0. This program was used to assess the vaccine's secondary structure for the presence of coils, -helixes, -sheets, and disordered domains^43^.

### Codon optimization and in silico cloning preparation

The reverse translation and codon optimization of the TEV was carried out using the Java Codon Adaptation Tool (JCat). This step was evaluated using the results of the percentage of GC content and the Codon Adaptation Index (CAI), and it was carried out for expression in an *E. coli* O6:K15:H31 (strain 536/UPEC). CAI reveals the propensity of an organism to express a heterologous gene. Successful optimization is defined as a CAI above 0.80 and a GC content between 30 and 70%, respectively^44^. The *NcoI* and *XhoI* restriction sites were used to insert the enhanced nucleotide sequence's N- and C-termini, respectively. The completed sequence was added to the pET28a (+) vector and evaluated by SnapGene for viability according to predictions.

### Tertiary structure prediction, refinement, and validation of the TEV

The TEV 3D model was constructed using the I-TASSER tool. The 3D model's accuracy was determined according to the confidence score (C-score). The C-score typically falls between -5 and 2, with a higher value denoting higher quality^45^. The constructed TEV model was refined using the Galaxy Refine tool to improve the quality of the 3D structure. This server reconstructs the side chains and repacks them using molecular dynamics modelling^46^.

The 3D structure of the predicted vaccine construct was verified using the PROCHECK service. The Ramachandran plot, which displays the fraction of amino acids in preferred, allowed, and banned zones based on the dihedral angles phi (φ) and psi (ψ) of each amino acid is the output of this server^47^. For additional validation, the ProSA online tool and ERRAT were used^48,49^.

### Molecular docking of the TEV with TLR4 and HLA

The ClusPro 2.0 tool was used to evaluate the potential of TEV docking with MHC and TLR immune receptors. The PDB files of MHC-II (PDB Id: 1H15 for HLA-DRB1), and TLR4 (PDB ID: 3FXI) were downloaded from the PDB database^50^. Furthermore, before docking between TLR4 and TEV, the 3D crystal structure of TLR4 was processed using the UCSF Chimera software. Then, non-amino acid molecules such as ligand, ions, and solvent water were removed from it, and subsequently polar hydrogen atoms were added to it^34,35^. The docked complexes presented by ClusPro 2.0 server with the lowest energy were chosen and then checked for protein–protein interaction using PyMOL software.

### Molecular dynamic simulation

The molecular dynamic simulation was carried out using the iMODS tool to examine the stability and physical movements of the TLR4-TEV and HLADRB1-TEV docked complexes^51^. This tool can predict the collective motions of proteins using normal mode analysis (NMA) in internal (dihedral) coordinates. The iMODS tool calculates the vaccine receptor complex’s deformability, eigenvalues, variance, covariance map, B-factor, and elastic network^51,52^.

### Ethical approval

This article does not contain any studies with human participants or animals performed by any of the authors.

## Results

### The protein sequence retrieval and analysis

The Sal k 1 allergen of *S. Kali* consists of 399 amino acids as follows: QPIPPNPAELESWFQGAVKPVSEQKGLEPSVVQAESGGVETIEVRQDGSGKFKTISDAVKHVKVGNTKRVIITIGPGEYREKVKIERLHPYITLYGIDPKNRPTITFAGTAAEFGTVDSATLIVESDYFVGANLIVSNSAPRPDGKRKGAQASALRISGDRAAFYNCKFTGFQDTVCDDKGNHLFKDCYIEGTVDFIFGEARSLYLNTELHVVPGDPMAMITAHARKNADGVGGYSFVHCKVTGTGGTALLGRAWFEAARVVFSYCNLSDAVKPEGWSDNNKPAAQKTIFFGEYKNTGPGAAADKRVPYTKQLTEADAKTFTSLEYIEAAKWLLPPPKV.

**Figure 1:**
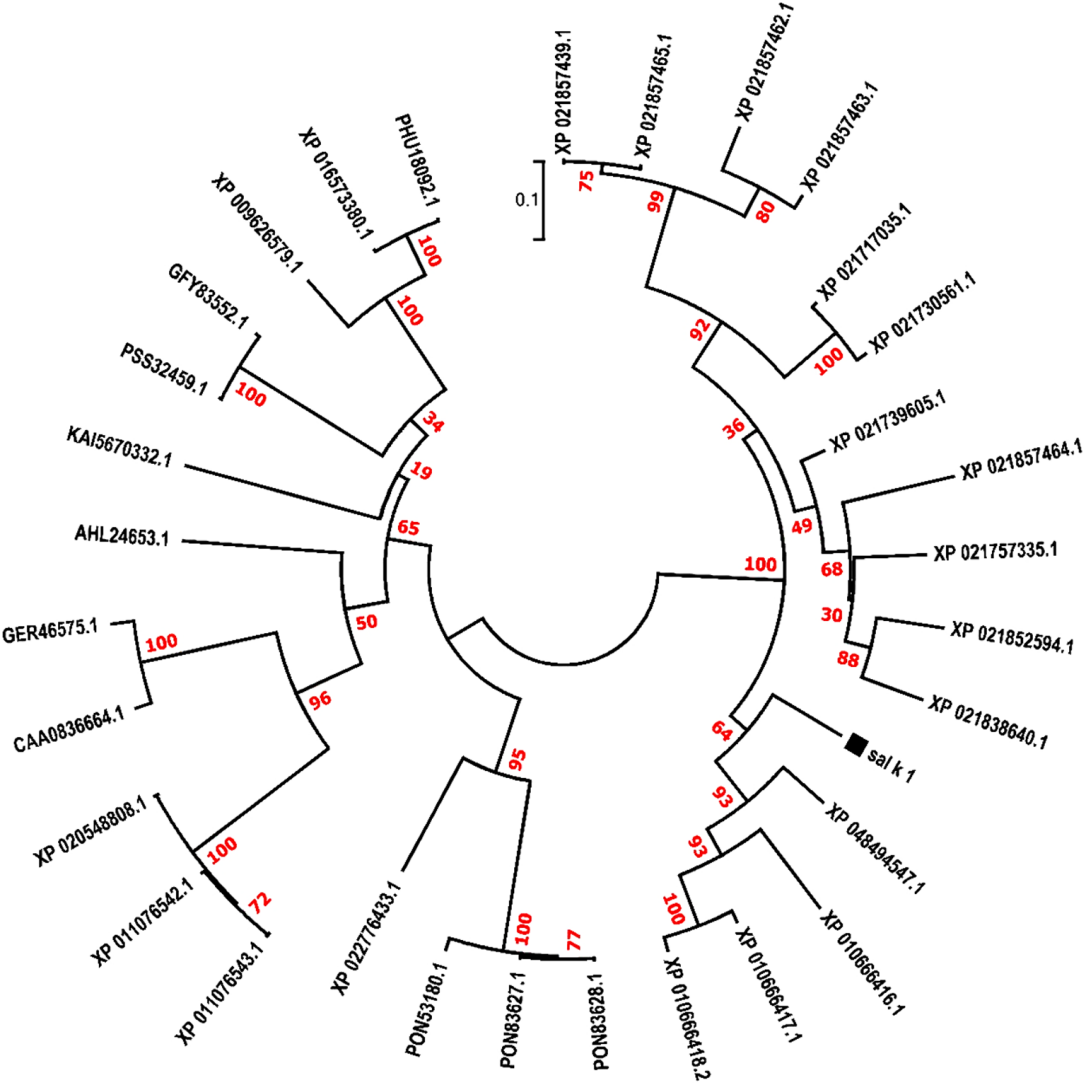
The phylogenetic relationship of the Sal k 1 allergen amino acid sequence was constructed by the ML method with 1000 bootstrap replicates.

The results of Pfam v29.0 and InterPro v56.0 showed that Sal k 1 belongs to the pectin esterase family. Moreover, the results of TMHMM Server 2.0 indicated that Sal k 1 protein was located outside of the cell membrane and had no transmembrane helices. Phylogenetic analysis was performed to determine the relationships between Sal k 1 and its homologous sequences. The evolutionary tree inferred by the maximum likelihood (ML) method has been indicated in Fig. 1.

### Physicochemical and secondary structure analyses

**Figure 2:**
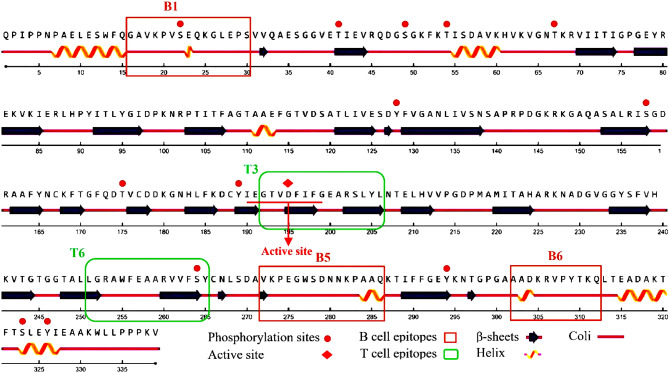
Sequence and secondary structure analyses of Sal k 1.

Investigation of the physicochemical properties of Sal k 1 using the ExPASy tool indicated that its molecular weight, hypothetical pI, instability index, aliphatic index, grand average of hydropathicity (GRAVY), and the total positive and negative charges were 36.79 kDa, 7.75, 24.08, 74.54, − 0.313, 40 and 39, respectively. The total number of atoms was 5163. Moreover, Sal k 1 had a functional pectin esterase motif PS00503 in the 190–199 (IEGTV**D**FIFG) position. Phosphorylation sites, including five Ser (22, 49, 158, 264 and 323), four Thr (41, 54, 67 and 175), and four Tyr (128, 189, 294, and 326) residues were predicted as it has been shown in Fig. 2.

Furthermore, the secondary structure prediction of Sal k 1 with PSIPRED identified eight α-helices (10.32%), twenty-one β- sheets (33.92%), and thirty-six random coil (55.65%) in Sal k 1. Alternatively, NetSurfP v2 predicted eight helixes (10.32%), twenty-two β-sheets (33.62%), and thirty-one random coils (56.05%), in Sal k 1 as it has been shown in Fig. 2.

In this investigation, random coils exhibited the highest number of residues followed by β-sheets and α-helixes. The lowest and the highest numbers of residues were found in the α-helixes and the random coils of Sal k 1, respectively (Fig. 2).

### Prediction of B lymphocyte epitopes

The amino acid sequence of Sal k 1 protein was subjected to Karplus & Schulz Flexibility, Emini surface accessibility, Chou & Fasman BetaTurn, Parker Hydrophilicity, Kolaskar and Tongaonkar antigenicity methods in Bcepred and IEDB tools to predict the binding to B cell (Table 1). From all the predicted B-cell epitopes, only three epitopes, i.e. **GAVKPVSEQKGLEPS**, **VKPEGWSDNNKPAAQ,** and **AADKRVPYTKQ**, were selected for further processing (Table 2). The predicted linear B-cell epitopes and their antigenicity, flexibility, degrees of accessibility, hydrophilicity, and the beta-turn prediction score have been summarized in Table 2.

**Table 1:** The predicted linear B cell epitopes of Sal k 1 allergen.

No	Start	End	Epitopes
1	16	30	**GAVKPVSEQKGLEPS**
2	27	37	LEPSVVQAESG
3	52	67	FKTISDAVKHVKVGNT
4	138	152	NSAPRPDGKRKGAQA
5	272	286	**VKPEGWSDNNKPAAQ**
6	302	312	**AADKRVPYT KQ**

*Epitopes with appropriate physicochemical properties have been shown in bold letters.

**Table 2:** The physicochemical prediction score for each residue of B cell epitopes from Sal k 1 allergen.

No	B cell epitope	Emini surface accessibility score (threshold = 1.000)	Parker hydrophilicity score (threshold = 1.835)	Karplus and Schulz flexibility score (threshold = 1.003)	Chou and Fashman beta turn score (threshold = 0.99)	Kolaskar and Tongaonkar antigenicity score (threshold = 1.024)
**1**	G	0.513	0.88	1.005	0.896	1.036
	A	1.000	1.243	0.994	0.976	1.06
	V	1.002	2.029	1.004	0.961	1.102
	K	0.931	2.1	1.019	1.026	1.101
	P	1.011	2.4	1.033	0.909	1.098
	V	2.125	2.957	1.048	0.954	1.091
	S	2.125	4.3	1.068	1.027	1.026
	E	1.36	4.3	1.081	1.106	1.018
	Q	1.511	2.686	1.084	0.973	1.045
	K	1.953	4.329	1.075	1.007	0.969
	G	1.744	3.7	1.056	1.02	0.976
	L	1.349	3.514	1.038	1.119	0.999
	E	0.501	2.129	1.026	1.05	1.052
	P	0.376	0.786	1.014	0.977	1.117
	S	0.789	0.829	0.995	0.894	1.137
**5**	V	1.545	4.357	1.014	1.046	1.024
	K	0.915	4.243	1.03	1.064	1.005
	P	0.953	1.386	1.035	0.993	1.008
	E	1.72	2.014	1.041	1.103	1.001
	G	1.436	3.971	1.034	1.24	0.927
	W	1.494	4.157	1.025	1.319	0.905
	S	1.387	4.857	1.037	1.324	0.864
	D	2.803	4.557	1.053	1.363	0.875
	N	4.122	4.043	1.076	1.357	0.902
	N	3.108	5.771	1.083	1.314	0.927
	K	1.88	5.143	1.068	1.204	0.934
	P	2.025	4.571	1.045	1.136	1.032
	A	2.518	4.386	1.021	1.057	1.030
	A	1.817	4.129	1.013	0.971	1.097
	Q	0.824	2.171	1.012	0.894	1.028
**6**	A	0.785	4.257	0.978	1.076	0.989
	A	1.553	4.557	0.992	0.994	0.962
	D	1.141	3.214	1.015	0.843	1.035
	K	1.747	3.214	1.025	0.966	1.035
	R	2.709	2.643	1.018	1.034	1.049
	V	2.342	3.086	1.003	1.077	1.027
	P	2.342	2.471	0.994	1.013	1.036
	Y	2.070	2.514	1.002	1.009	1.048
	T	2.300	0.901	1.015	0.957	1.102
	K	2.147	1.871	1.028	1.023	1.034
	Q	2.373	2.686	1.031	0.911	1.004

### Prediction of HTL epitopes and population coverage

The binding epitopes to specific MHC-II molecules in the protein sequence of Sal k 1 were predicted using the IEDB database. On the basis of low IC50 values, low percentile rank, and reactive with diverse MHC-II alleles only 9 potential peptides were selected for further processing. Peptides with the ability to bind to a larger number of alleles are identified as the most suitable cases due to their potential to elicit a potent protective response. The list of the most promising HTL epitopes and their correspondent binding MHC II alleles have been shown in Table 3. In order to design the TEV, population coverage was analyzed for epitopes binding to diverse alleles of MHC-II. High population coverage of the vaccine is important because many people can benefit from it with just one proper vaccine. The result of combining these methods with MHC- II limitation for the whole world, European, and Southwest Asia population with the selected MHC- II alleles has been indicated in Table 3.

**Table 3:** The most potential HTL epitopes with interacting MHC-II alleles and population coverage.

No	CD4 + T Cell epitopes	Length	Start	End	MHC-II binding alleles	*Percentile rank*	*Smm IC50*	Population coverage		
								World	Europe	Southwest Asia
1	NRPTITFAGTAAEFG	15	101	115	HLA-DQA1*04:01/DQB1*04:02	0.23	159	79.44%	75.61%	76.82%
					HLA-DQA1*03:01/DQB1*03:02	1.3	418			
					HLA-DQA1*05:01/DQB1*02:01	3.2	571			
2	KNRPTITFAGTAAEF	15	100	114	HLA-DQA1*04:01/DQB1*04:02	0.26	155	82.76%	77.22%	77.92%
					HLA-DQA1*03:01/DQB1*03:02	1.5	464			
					HLA-DQA1*05:01/DQB1*03:01	7.2	71			
3	**GTVDFIFGEARSLYL**	15	192	206	HLA-DRB1*07:01	0.28	11	85.73	98.31%	84.82%
					HLA-DRB1*09:01	2	97			
					HLA-DRB1*01:01	5.4	30			
					HLA-DRB1*03:01	5.9	362			
					HLA-DRB5*01:01	8.5	59			
					HLA-DPA1*01:03/DPB1*04:01	9	454			
4	GANLIVSNSAPRPDG	15	131	145	HLA-DRB3*02:02	0.52	53	55.65%	48.36%	48.11%
					HLA-DRB1*13:02	1.4	5.4			
					HLA-DRB1*03:01	4.7	114.4			
					HLA-DRB1*09:01	6.4	581			
5	AKTFTSLEYIEAAKW	15	318	332	HLA-DPA1*01:03/DPB1*04:01	0.68	98	81.44%	97.02%	23.74%
					HLA-DPA1*03:01/DPB1*04:02	1.9	864			
					HLA-DPB1*02:01	3.4	345			
6	**LGRAWFEAARVVFSY**	15	251	265	HLA-DPA1*02:01/DPB1*14:01	0.76	100	91.74%	92.75%	88.43%
					HLA-DQA1*01:02/DQB1*06:02	0.97	79			
					HLA-DQA1*05:01/DQB1*03:01	3.3	37			
					HLA-DPB1*05:01	6.1	245			
					HLA-DRB1*09:01	6.6	208			
7	EFGTVDSATLIVESD	15	113	127	HLA-DQA1*04:01/DQB1*04:02	0.78	340	77.41%	7.86%	65.00%
					HLA-DQA1*01:02/DQB1*06:02	1.8	111			
					HLA-DQA1*03:01/DQB1*03:02	2	209			
8	GRAWFEAARVVFSYC	15	252	266	HLA-DQA1*01:02/DQB1*06:02	0.98	80	82.00%	81.00%	73.34%
					HLA-DPA1*02:01/DPB1*14:01	1.2	98			
9	AELESWFQGAVKPVS	15	8	22	HLA-DQA1*01:01/DQB1*05:01	3.4	847	31.46%	32.82%	35.61%

*HTL epitopes with high affinity to binding variant HLA alleles have been shown in bold letters.

### Docking analysis of HTL epitopes

**Figure 3:**
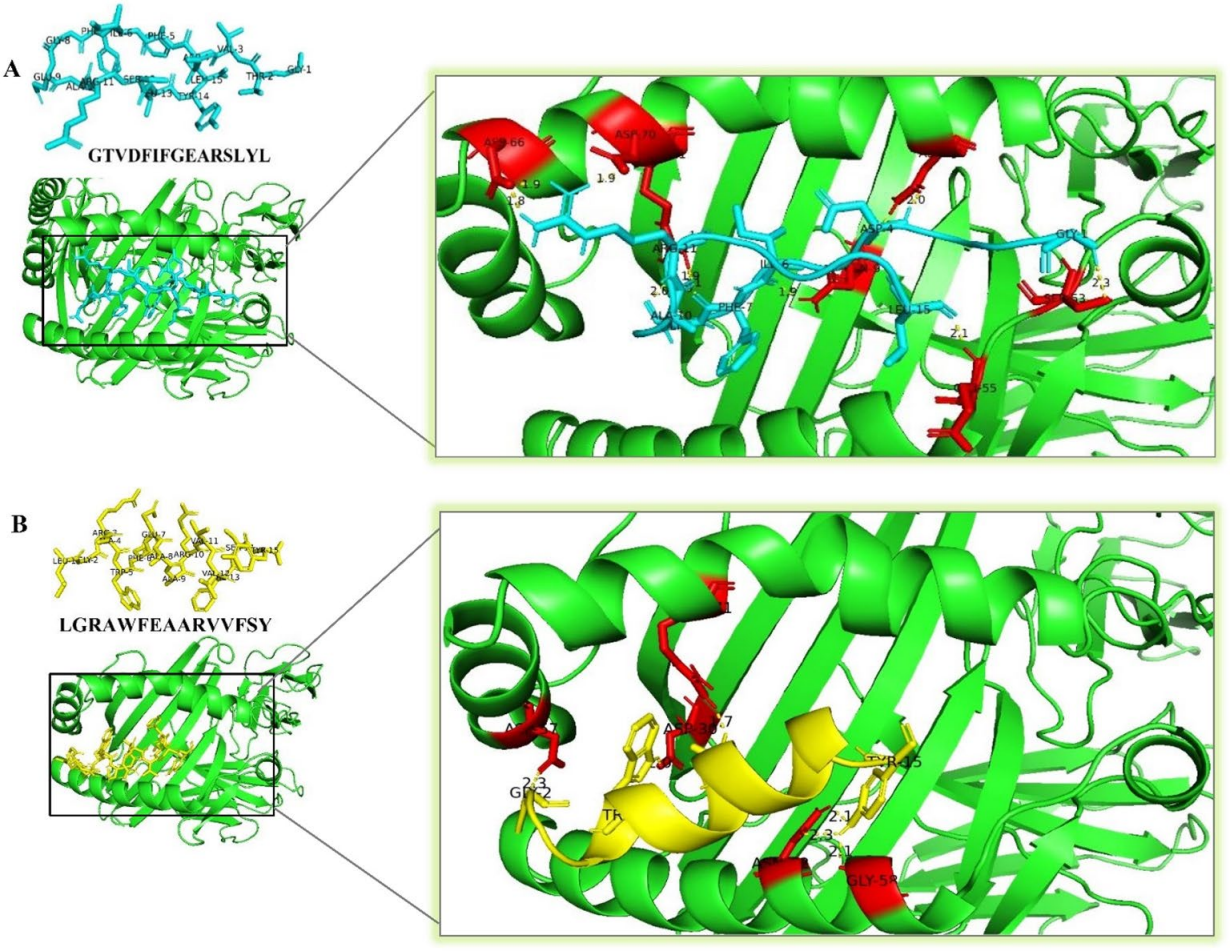
(**A**, **B**) docking of the predicted HTL epitopes with the HLA DRB1 (PDB: 1H15) molecule. Amino acid residues of HLADRB1 that interact with ligands have been shown in red color.

Using the PEP-FOLD 3 server, the 3D structures of the selected main epitopes of HTL (**GTVDFIFGEARSLYL**, and** LGRAWFEAARVVFSY)** were predicted (Fig. 3). Ten models were predicted for each epitope, and the model with the lowest energy was selected for subsequent investigation. The chosen structures were docked with the crystal structure of the human HLA.DRB1 (1H15) using ClusPro v2.0 and PyMOL tools (Fig. 3).

### Construction of the TEV

**Figure 4:**
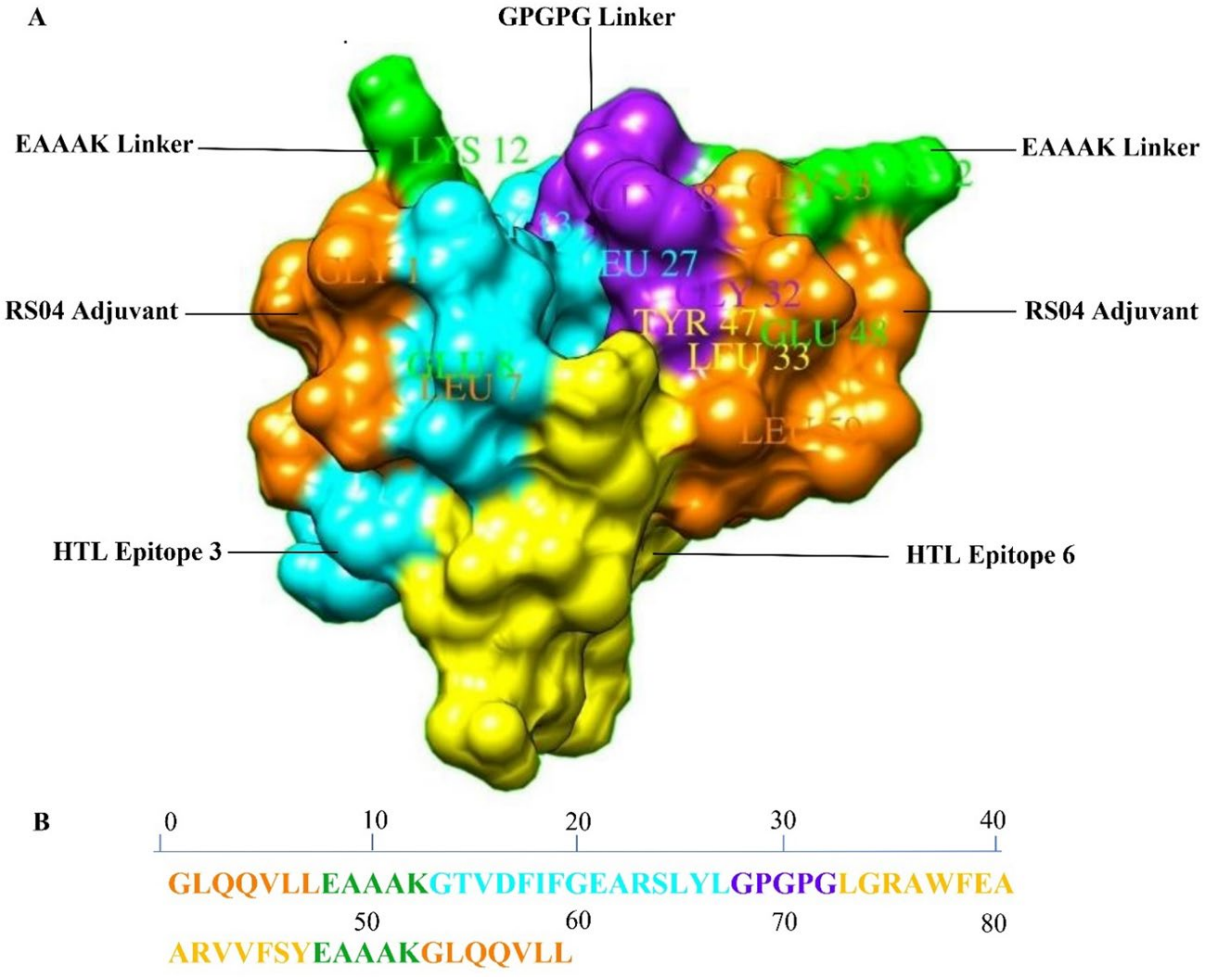
(**A**) Illustration of the 3D construct TEV of Sal k 1 in a space-filling model. (**B**) Sequence of the TEV of Sal k 1.

Epitopes three (**GTVDFIFGEARSLYL**) and six **(LGRAWFEAARVVFSY)** (Tabel 3) were selected from the predicted epitopes for HTL. Subsequently, they were connected together by the GPGPG linker. A 35 amino acids sequence was produced after epitope fusion. The adjuvant sequence with a length of 7 amino acids (LPS mimic peptide **RS04: GLQQVLL**) was fused to the N and C terminals of the vaccine sequence by an EAAAK linker. The final designed vaccine construct was comprised of 59 amino acids (Fig. 4). Based on the result obtained from BLASTp, the vaccine construct was not identical with the human proteome and human microbiome proteins.

### Antigenicity and safety of the constructed TEV

The probability of antigenicity predicted by ANTIGENpro was 0.108, which is indicative of its highly antigenic characteristics. Moreover, the vaccine models on both Algpred and ToxinPred tools were non-allergen and non-toxic, respectively.

### Physicochemical properties of the TEV

The Expasy ProtParam tool was used to predict different physicochemical features of the designed TEV. The final composition of the TEV was composed of 59 amino acids. The theoretical pI, molecular weight, and instability index of the vaccine construct were calculated to be 6.31, 6.252 kDa, and 21.6, respectively. The half-life of the vaccine was estimated to be 30 h in mammalian reticulocytes in vitro, more than 20 h in the yeast in vivo, and more than 10 h in *E. coli*, in vivo. The GRAVY score of the vaccine was 0.405, and its aliphatic index was calculated to be 107.63. A solubility score of 0.916673 indicated that the TEV was soluble after the expression in *E. coli*.

### Secondary structure properties of the TEV

**Figure 5:**
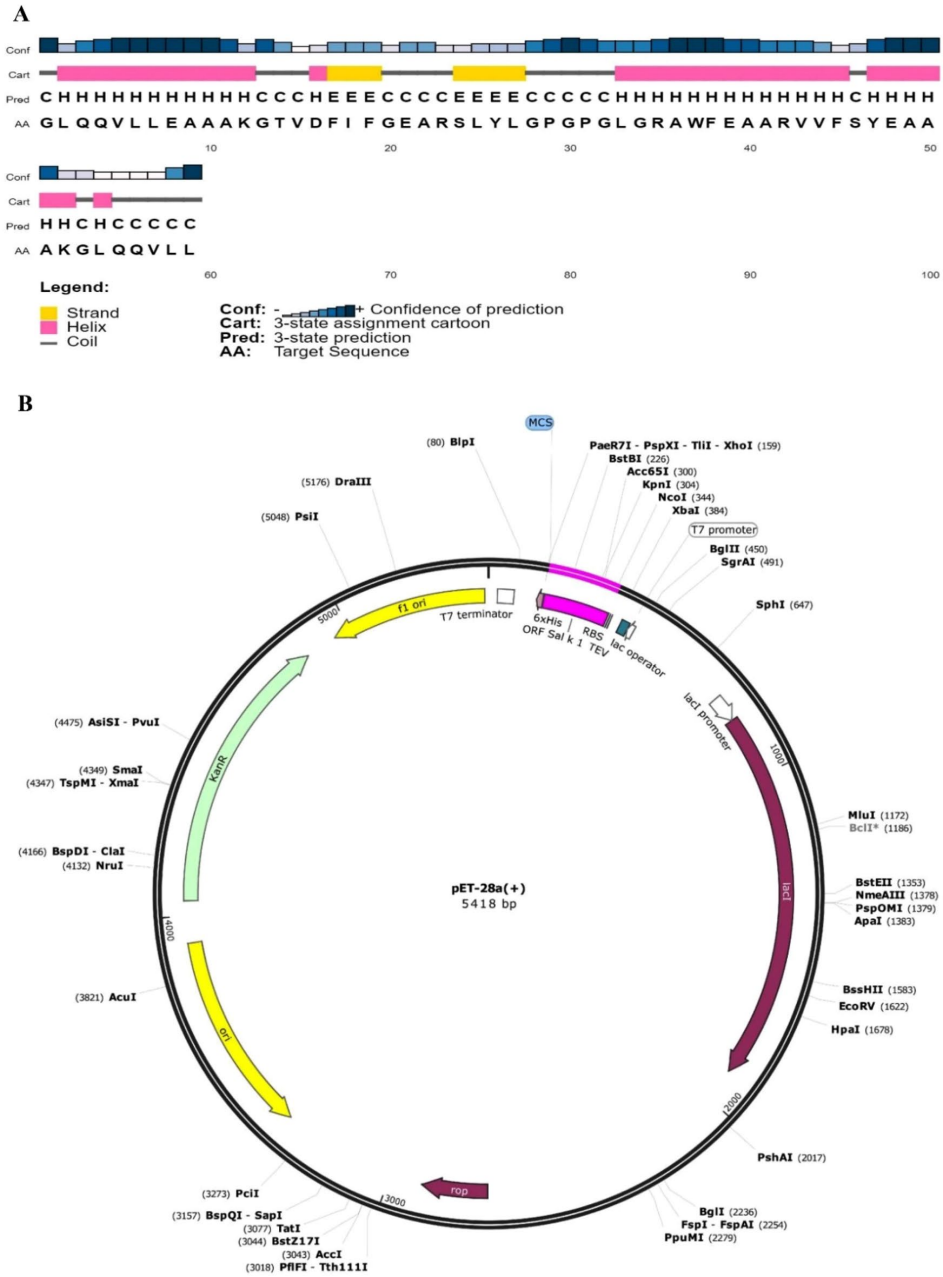
(**A**) The secondary structure TEV of Sal k 1. (**B**) In silico cloning of the TEV sequence of Sal k 1 into the pET28a (+). The pink colored region shows the ORF and coding sequence of Sal k 1 in the pET28a (+) plasmid structure.

The PSIPRED server predicted that the TEV contained 5.084% alpha-helix, 55.93% random coil, and 38.98% β-strand region (Fig. 5A).

### Codon optimization and in silico cloning

Reverse translation and codon optimization in the JCat tool produced a 177 bp nucleotide sequences for the constructed vaccine. The CAI values of the optimized sequences and GC contents were 1.0 and 55.36% respectively, indicating the desirable expression of the vaccines within *E. coli*. Subsequently, a recombinant plasmid was designed by inserting the vaccine sequence between *Nco*I and *Xho*I restriction sites in the pET28a (+) expression vector using the SnapGene software (Fig. 5B).

### Modeling the 3D structure, refinement, and validation of the TEV

**Figure 6:**
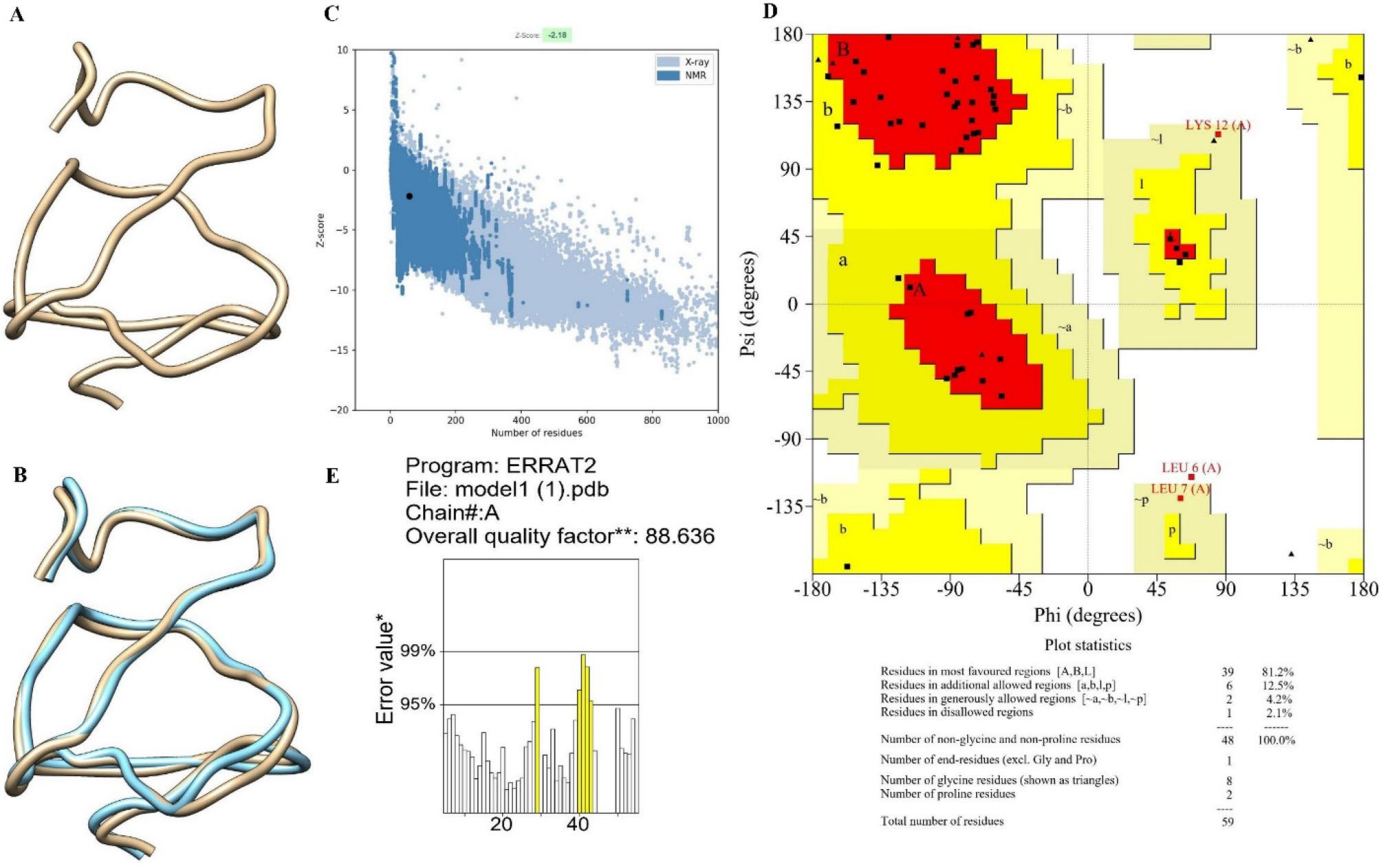
The 3D structure, refinement and validation of the TEV. (**A**) The 3D structure of the TEV was predicted by I-TASSER server. (**B**) the 3D structure of the TEV after refinement using Galaxy Refine server. (**C**) ProSA-web, with a Z score of -2.18. (**D**) The Ramachardan plot indicated that 81.2% of the residues were in the most favored region, 12.5% were in the additional allowed region, 4.2% were in the generously allowed region, and 2.1% were in the disallowed region. (**E**) The ERRAT quality factor was 88.636%.

Five models of the 3D structure of the TEV construct were produced by the I-TASSER server using the threading templates (PDB: 1gq8A, 1gq8A, 3uw0A, 1xg2A, 1xg2A, 1gq8A, 3grhA, 3grhA, 3grhA, and 3grh). The calculated C-score values for models 1–5 were − 1.55, − 1.87, − 2.66, − 3.79, and − 4.67, respectively. The C-score is normally in the range of (− 5, 2), where high score shows high confidence in the predicted model. According to these criteria, the.

best model (C-score: − 1.55) was chosen for further investigation. Furthermore, this model had the TM score and root-mean-square deviation (RMSD) score of 0.52 ± 0.15 and 6.0 ± 3.7 Å, respectively (Fig. 6A). TM-score is a proposed scale for measuring the structural similarity between two structures^53^. The Galaxy Refne tool was used to improve the consistency of the vaccine model. Five models were generated from the refinement of the primary “crude” vaccine model. Model 4 was the most important model based on structure qualities, i.e. GDT-HA (0.9364), RMSD (0.491), and MolProbity (1.90), clash value 5.5), Poor rotamers (0.0) and Rama favored (87.7) (Fig. 6B). This model was chosen for further analysis. The Ramachandran plot analysis was used to validate the refined 3D structure of the vaccine construct. The results showed that 81.2% of the residues were in the best region, 12.5% were in the additional allowed region, 4.2% were in generously allowed regions, and 2.1% were in the disallowed region (Fig. 6C). The ProSA-web evaluated the Z score of the vaccine's model to be − 2.18 (Fig. 6D). Furthermore, the refinement model had an overall quality factor of 88.636% with ERRAT ((Fig. 6E).

### Molecular docking of the TEV with TLR4 and HLADR

**Figure 7:**
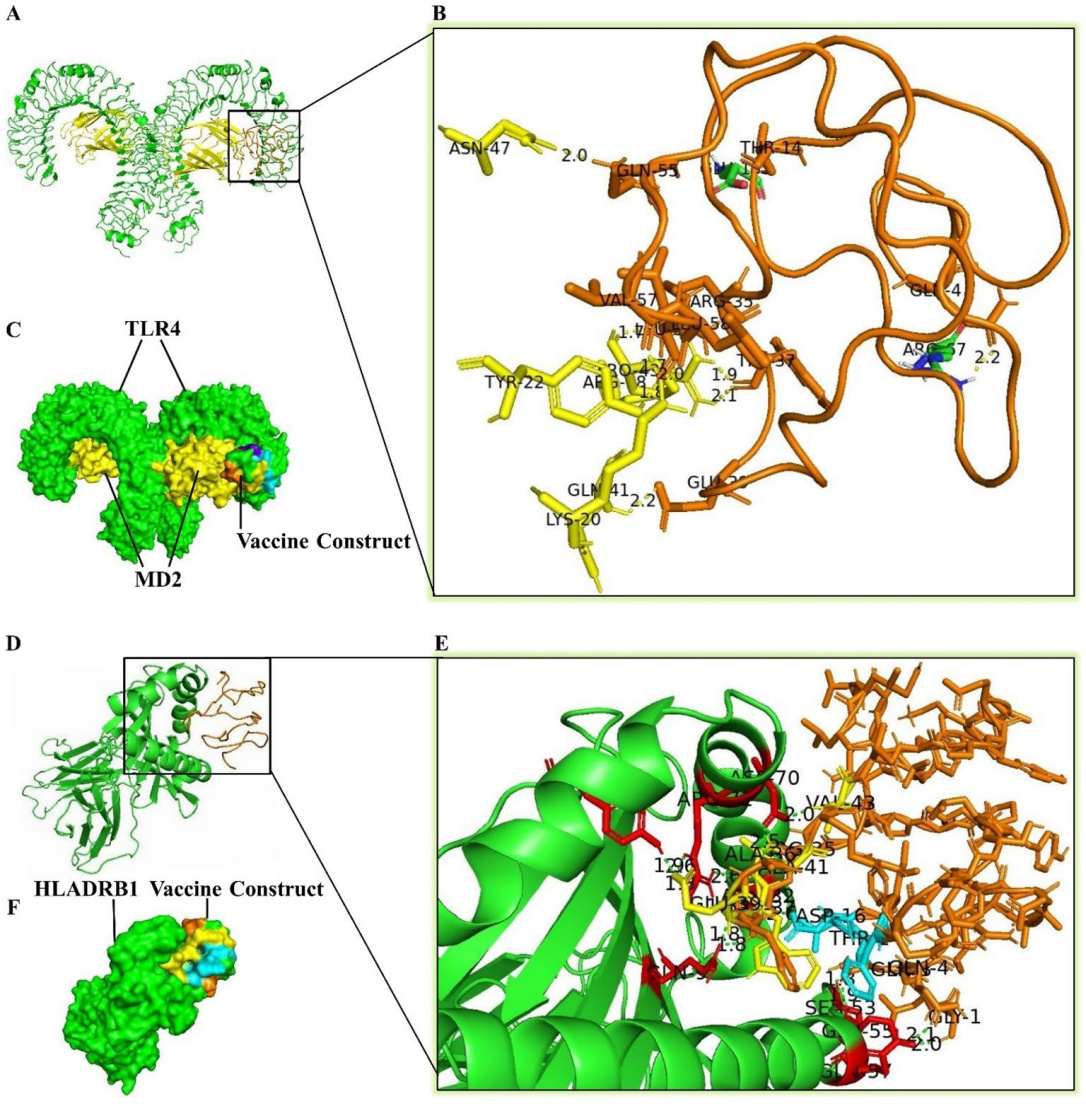
Molecular docking between the TLR4 (**A**–**C**), and HLADRB1 (**D**–**F**) with the TEV, T cell epitopes 3 and 6 interacting with HLADRB1 have been shown in cyan and yellow, respectively. Amino acid residues of HLADRB1 that interacted with TEV have been shown in red color.

Binding the TEV construct to the human immune receptors was evaluated using PyMOL and ClusPro 2.0 tools (Fig. 7A–F). The results showed that the TEV had a high affinity to bind TLR4 receptor with − 823.3 kcal/mol (Fig. 7A–C). Furthermore, it was shown that the TEV had a high affinity to bind in a grave of HLADRB1 with − 933.9 kcal/mol (Fig. 7D–F). The use of the PyMOL tool indicated that different residues of the TEV could interact with TLR4 and HLADRB1 (Table 4).

**Table 4:** The list of amino acids residues involved in hydrogen bonds between the TEV and TLR4 (chain B) and HLADRB1 (the region variable of α and β chains).

HLADR β chain	TEV	Bond length *Å*	HLADR α chain	TEV	Bond length Å	MD2	TEV	Bond length Å	TLR chain B	TEV	Bond length Å
His28	Thr14	2.7	Ala61	Gln75	2.1	Arg68	Trp37	1.9	Arg67	Glu4	2.2
Asn82	Val57	2.0	Met36	Arg55	1.9	Gln41	Glu39	2.2	Glu135	Thr14	1.9
Arg71	Val15	1.7	Gln57	Arg55	2.0	Pro43	Arg35	1.7			
	Asp16	2.0	Arg71	Ala36	2.6	Lys20	Val57	1.7			
				Trp37	1.8		Leu59	2.0			
Asp70	Phe19	2.1	Tyr47	Glu39	1.9	Tyr22	Val57	1.8			
Tyr60	Lys12	1.8	Asp70	Ala41	2.5	Asn47	Gln55	2.0			
Gln64	Lys12	1.9		Val43	2.0	–	–	–			

### Molecular dynamic (MD) simulation

**Figure 8:**
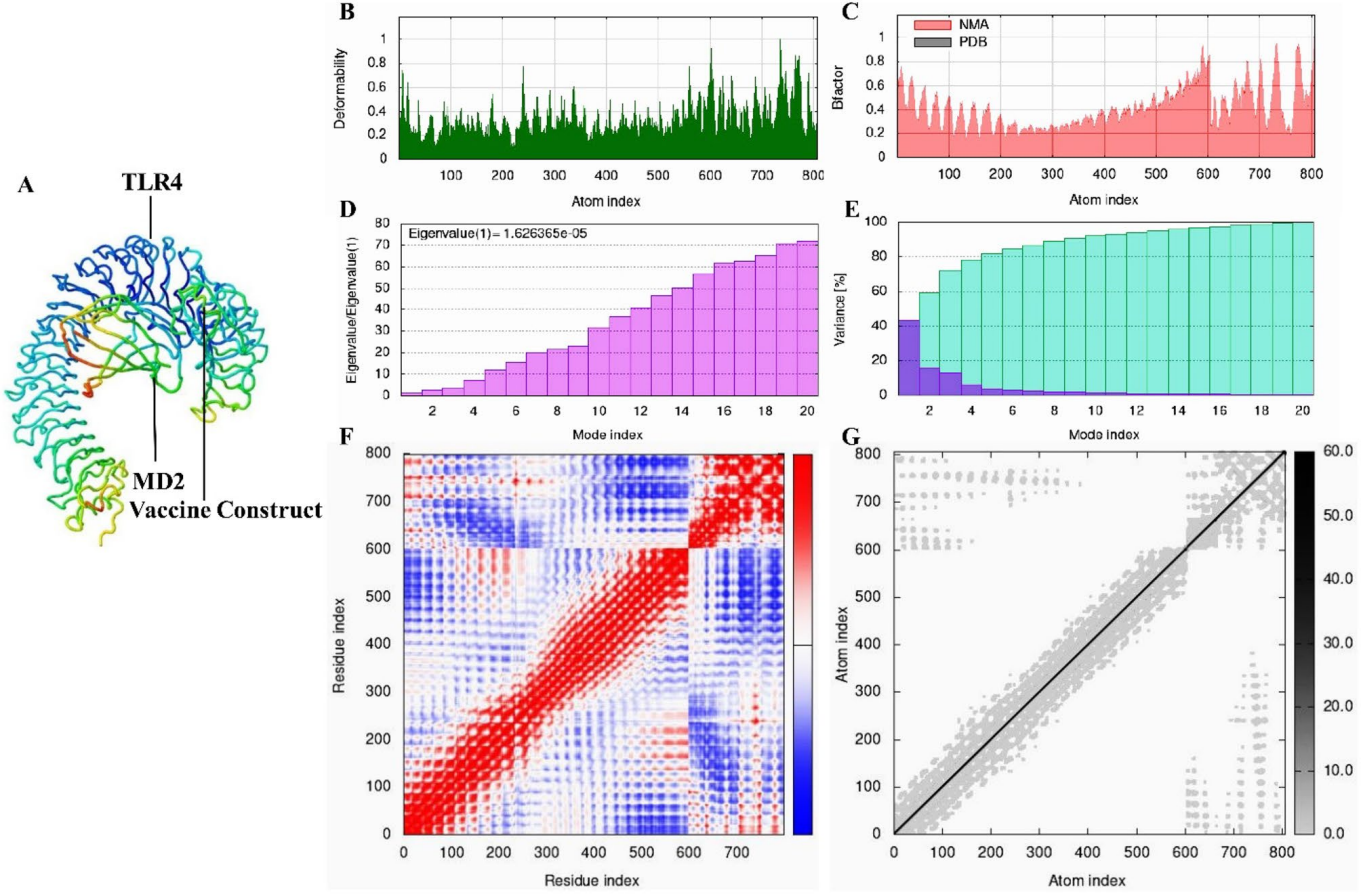
The results of iMODS simulations of molecular dynamics (MD) for TLR4-TEV. (**A**) Areas of low mobility (blue) to high mobility (red) have been highlighted in the TLR4 and vaccine docking complex normal mode analysis (NMA). (**B**) deformability. (**C**) B-factor. (**D**) Eigenvalues (a lower value means easier deformation). (**E**) Variance (red color: individual variances and green color: cumulative variances). (**F**) Covariance map (red: correlated, white: uncorrelated, blue: anti-correlated). (**G**) Elastic network (darker regions mean stiffer regions)**.**

**Figure 9:**
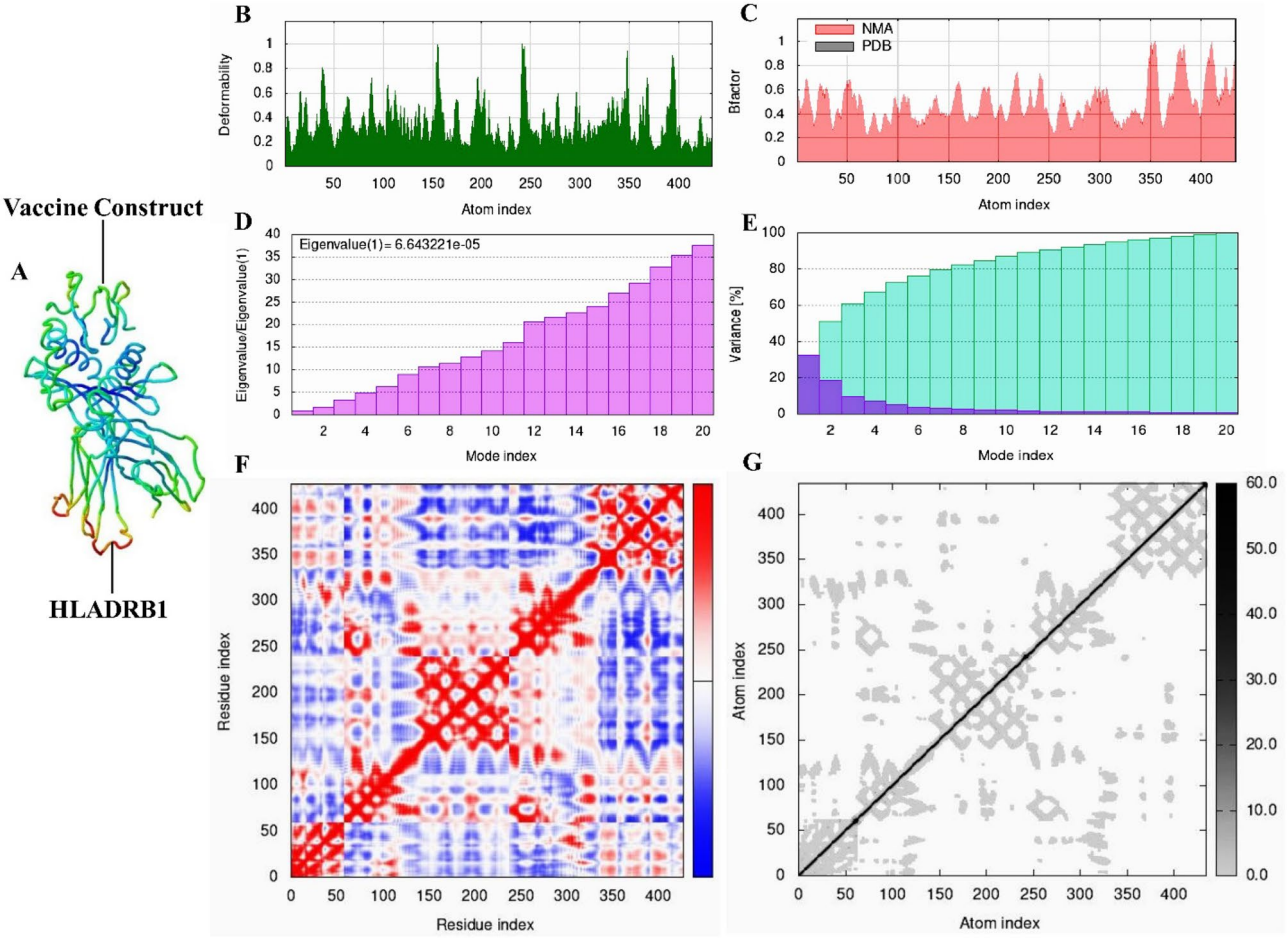
The results of molecular dynamics simulations for HLADRB1-TEV in iMODS. (**A**) Areas of low mobility (blue) to high mobility (red) have been highlighted in the HLADRB1 and vaccine docking complex normal mode analysis (NMA). (**B**) deformability. (**C**) B-factor. (**D**) Eigenvalues (Low value indicates simpler deformation). (**E**) Variance (red color: individual variances and green color: cumulative variances). (**F**) Covariance map (red: correlated, white: uncorrelated, blue: anti-correlated). (**G**) Elastic network (darker regions mean stiffer regions).

We used MD simulations for the iMODS tool to examine the stability and motions of the docked complexes. Slow dynamics of the docked complexes were investigated, and their large-amplitude conformational variations were shown using normal mode analysis (NMA). Figures 8A and 9A show the NMA of the docked complexes of the TLR4-TEV and HLADRB1-TEV, respectively. The mobility features of the docked proteins are determined by the deformability and B-factor. The peaks that correlate to the regions in the proteins with deformability are shown by the deformability and B-factors of the TLR4-TEV and HLADRB1-TEV complexes, where the highest peaks reflect the regions of high deformability. The B-factor diagrams allow for a comparison of the complexes' NMA and PDB fields (Figs. 8C and 9C). The deformability and B‐factor of TLR4‐TEV and HLADRB1‐TEV complexes have been illustrated in Figs. 8 and 9, respectively. Eigenvalue, which is a crucial parameter of a stable structure, must be high to have a stable complex^52^. The eigenvalue rates for the simulated docking complexed TLR4-TEV and HLADRB1-TEV were 1.626365e-05 and 6.643221e-05 respectively. These rates are significantly higher for structural stability (Figs. 8D and 9D). The covariance matrix of the TLR4‐TEV and HLADRB1‐TEV complexes shows the correlations among residues in a complex. The white color in the matrix represents uncorrelated motions, while the red color shows a good correlation between the residues. Moreover, the blue color exhibits anticorrelations. The higher the correlation, the better the complex. The relationships between the atoms have been shown in the elastic maps of the docked proteins, where the stiffer regions have been exhibited by the darker grey areas (Figs. 8G and 9G).

## Discussion

Over the past few decades, atopic illnesses in humans such as allergic rhinitis, asthma, and atopic dermatitis have become more common^2,54^. The increased ability to diagnose these diseases is a major concern for the global medical community^55^. Allergen specific immunotherapy (AIT) with an extract containing intact allergens is usually accompanied by a severe reaction in allergic patients, which can even cause anaphylactic shock^56,57^. Peptides prepared from allergenic substances such as tree pollens and venom insects have been used in human clinical studies and experimental animal models^58^. The use of T cell epitopes can cause tolerance to allergens and also prevent allergic reactions caused by IgE antibodies during conventional AIT^59^. Furthermore, it has been shown that when a high-dose specific allergen T cell epitope is used for treatment. It causes anergy and elimination in allergen-specific Th2 cells, and also induces Treg with IL-10 production^60^. Specific epitopes of CD4+ T lymphocytes are central to T cell-based epitope immunotherapy. Recognition of antigenic epitopes by T cells depend on their presentation along with the highly polymorphic molecules HLADR, HLADQ and HLADP. For this reason, epitopes in T cell-based epitope immunotherapy should be attached to different alleles of HLA molecules that can be used in different populations^61^. Based on this criterion, in this study, two T cell epitopes from regions 192–206 (**GTVDFIFGEARSLYL)** and 251–265** (LGRAWFEAARVVFSY)** were selected, which were bound to various alleles of HLA molecules with high binding affinity. The predicted T cell epitopes were examined for allergenicity, toxicity, and antigenicity. Also, to prevent allergic reactions caused by IgE antibodies, these T cell epitopes were chosen so that they did not have any overlapping with the epitopes predicted to bind to B cells. In the present study, we used GPGPG linkers to link HTL epitopes, as it had already been done in previous studies^62,63^. The GPGPG linker promotes epitope presentation, while reducing the development of junctional epitopes^64^. Moreover, in this study, RS04 adjuvant was used as a TLR4 agonist in the structure of the TEV. RS04 is a synthetic peptide that mimics LPS by interacting with TLR4. It has been reported that this adjuvant, unlike alum and other adjuvants, has no side effects and can potentially stimulate immune responses through interacting with TLR4^37^. Following performing the analysis by the PyMOL tool, it was observed that the amino acid residues of Gln55, Val57, and Leu59 related to RS04 in the structure of the TEV interacted well with the amino acid residues of the B chain of the TLR4 molecule (Fig. 7A–C and Table 4). This interaction had an effective role in binding the TEV and stimulating the immune system. The HTL epitopes identified in the Sal k 1 allergen have a remarkable binding affinity with DRB*0101 alleles. A large proportion of human populations have this gene, which causes the immune system to react quickly and effectively. After checking by the PyMOL tool, it was observed that several amino acid residues in the TEV structure interacted with the amino acid residues of the HLADRB1 molecule (Fig. 7D–F and Table 4), and this indicated the effective efficiency of the constructed TEV. The TEV instability index was calculated to be 21.6. If this number is less than 40, the protein is thought to be stable^24^. In mammalian reticulocytes, the half-life of our TEV was found to be 30 h. This finding suggests that our TEV could be exposed to the immune system for a longer period of time, the same as the vaccine created by Fanuel et al.^65^. The TEV’s aliphatic index was determined to be 107.63, indicating that it was thermostable^65^. The GRAVY value of the TEV was 0.405, and positive values for this metric denoted a hydrophobic property of the vaccine^66^. GRAVY was found to be 0.252 in previous studies^67^. However, because of the hydrophobic nature of the vaccine, it appears that micelles must be used to boost vaccine interaction inside the polar environment of the body. TLR4-TEV and HLADRB1-TEV docked complexes were also subjected to MD simulation to determine the vaccine construct’s stability. The results of the iMODS revealed that TLR4-TEV and HLADRB1-TEV complexes were stable (Figs. 8 and 9). The TEV sequence had a CAI value of 1.0 and a GC content of 55.36%. The results of this section were satisfactory for two reasons. First, CAI values more than 0.8 are thought to be excellent for expression in the host organism^68^. Second, it has been indicated that improved expression requires a GC content between 30 and 70%^68^. In this study, we designed a T-cell epitope-based vaccine. This vaccine was developed based on many in silico investigations. This approach can contribute to the rapid prediction of a vaccine's potential prior to experimental testing. The vaccine was developed based on HTL epitopes from Sal k 1, which is an allergenic protein found in *S. kali* pollen grains. Antigenicity and safety of the TEV were demonstrated via the evaluation of its allergenicity, solubility, and toxicity. Furthermore, the TEV designed in this study should not cause autoimmune diseases in humans due to the lack of any similarity and identity with human and microbiota proteome. Moreover, the interaction of the TEV with TLR4 and HLADRB1 demonstrated its ability to stimulate innate and adaptive immune responses. The present study is the first step in the TEV vaccine production procedure for Sal k 1 allergen. Further in vivo studies are required to investigate other aspects of this procedure.

## Data Availability

The datasets generated and/ or analyzed during the current study are available within the manuscript and in NCBI (https://www.ncbi.nlm.nih.gov/) with their accession numbers P83181, AAT99258, AAX11262, and AAX11262 in this manuscript.
